# Thinking Back about a Positive Event: The Impact of Processing Style on Positive Affect

**DOI:** 10.3389/fpsyt.2015.00003

**Published:** 2015-03-09

**Authors:** Sabine Nelis, Emily A. Holmes, Rosa Palmieri, Guglielmo Bellelli, Filip Raes

**Affiliations:** ^1^Faculty of Psychology and Educational Sciences, KU Leuven – University of Leuven, Leuven, Belgium; ^2^MRC Cognition and Brain Sciences Unit, Cambridge, UK; ^3^Department for Clinical Neuroscience, Karolinska Institutet, Stockholm, Sweden; ^4^Libera Università Maria Ss. Assunta (LUMSA) University, Taranto, Italy; ^5^EMPEA: Clinical Research Centre for Cognitive-Behavioral Psychotherapy, Bari, Italy; ^6^University of Bari Aldo Moro, Bari, Italy

**Keywords:** memory, processing style, positive, recall, mental imagery, abstract/verbal processing

## Abstract

The manner in which individuals recall an autobiographical positive life event has affective consequences. Two studies addressed the processing styles during positive memory recall in a non-clinical sample. Participants retrieved a positive memory, which was self-generated (Study 1, *n* = 70) or experimenter-chosen (i.e., academic achievement, Study 2, *n* = 159), followed by the induction of one of three processing styles (between-subjects): in Study 1, a “concrete/imagery” vs. “abstract/verbal” processing style was compared. In Study 2, a “concrete/imagery,” “abstract/verbal,” and “comparative/verbal” processing style were compared. The processing of a personal memory in a concrete/imagery-based way led to a larger increase in positive affect compared to abstract/verbal processing in Study 1, as well as compared to comparative/verbal thinking in Study 2. Results of Study 2 further suggest that it is making unfavorable verbal comparisons that may hinder affective benefits to positive memories (rather than general abstract/verbal processing *per se*). The comparative/verbal thinking style failed to lead to improvements in positive affect, and with increasing levels of depressive symptoms it had a more negative impact on change in positive affect. We found no evidence that participant’s tendency to have dampening thoughts in response to positive affect in daily life contributed to the affective impact of positive memory recall. The results support the potential for current trainings in boosting positive memories and mental imagery, and underline the search for parameters that determine at times deleterious outcomes of abstract/verbal memory processing in the face of positive information.

The regulation of positive affect and the processing of positive material are gaining attention in the context of mood disorders [e.g., Ref. (1–6)]. Depression, for instance, is not only characterized by increased negative affect but also by anhedonia, which refers to reduced positive affect or reduced pleasure in daily activities [(7), p. 160]. Besides targeting negative affect, therefore, promoting positive affect, targeting responses to positive stimuli may enhance interventions for depression [e.g., Ref. (1, 2, 8, 9)]. One specific type of positive stimuli is positive memories. In a review on blunted positivity in depression, Dunn (2) has mentioned the need for more research on positive memories. For instance, positive memories in formerly depressed individuals have decreased vividness compared to never-depressed controls (10). Further, Joormann et al. (11) showed that, whereas never-depressed individuals are able to repair their sad mood by retrieving positive memories, this strategy is unsuccessful in formerly and currently depressed individuals. This suggests that recollection of positive memories may lead to multiple affective outcomes. Therefore, knowledge of relevant thought patterns is crucial to understand the relationship between positive memory recall and affect.

For negative stimuli, the way a negative event is processed, for instance, concrete/imagery-based vs. abstract/verbally based processing, influences subsequent affective responding [e.g., Ref. (12–14)]. A concrete/imagery-based processing style involves a situation-specific sensory processing of the event, focused on the experience of the moment (e.g., focusing on the sights and sounds). Abstract/verbal processing of a certain situation is more analytical and involves verbal thinking about the meanings, causes, and consequences or implications of an event.

More recently, research on the impact of processing styles was broadened from the domain of negative affect or situations to positive stimuli and positive memories [e.g., Ref. (5, 15, 16)]. When processing hypothetical positive situations, for instance, concrete/imagery-based processing instructions improve mood [e.g., Ref. (5, 15, 17)]. Mental imagery, therefore, is also referred to as an “emotional amplifier” [e.g., Ref. (18)]. By contrast, participants in these experiments who were instructed to think verbally (and abstractly) about positive scenarios do not show mood improvement. Moreover, it is sometimes found that verbal thoughts even lead to mood deterioration in the face of positive information (5, 15).

With regard to positive memories, Bryant et al. (19) demonstrated that daily sessions of imagining positive memories increase the amount of time that individuals report to feel happy. However, this study did not include a non-imagery control for positive memories. Positive memories have also been investigated in clinical samples as a strategy to repair sad mood: Werner-Seidler and Moulds (16) demonstrated that concretely processing a joyful memory may indeed help to reduce sad feelings in (previously) depressed individuals (16). In contrast, an abstract thinking style focused on causes, meanings, and consequences as well as on making comparisons between a past happy memory and the current life did not improve sad mood in (previously) depressed persons [this was not entirely replicated when participants retrieved a self-defining memory (20)].

Interestingly, it has been proposed that not all types of abstract/verbal processing cause mood deterioration. For instance, Holmes et al. [(5); Experiment 2] showed that especially making active verbal comparisons could be responsible for mood deterioration after verbal processing instructions for positive scenarios. Thus, even in non-clinical samples, hypothetical “overly” positive situations (i.e., ones that incurred unfavorable comparisons) could be stressful in that, in your own life, events may not end up that positive. In addition to the detrimental effect of unfavorable comparisons on positive affect, we also suggest dampening as a potential moderator of the impact of abstract/verbal processing of positive events on positive affect. Interestingly, some individuals are characterized by a “*dampening response” style* to positive affect (21). Dampening is a particular cognitive response style or emotion regulation strategy, defined as “the tendency to respond to positive moods states with mental strategies to reduce the intensity and duration of the positive mood state” [(21), p. 509]. Examples of dampening thoughts in response to positive affect are: “I do not deserve this,” “you know, these feelings won’t last,” or “thinking about those times that did not go well.” Dampening can be measured by the Responses to Positive Affect questionnaire (RPA) (21, 22) and is positively associated with depressive symptoms [e.g., Ref. (23)]. Dampening can be regarded as a type of analytical thinking, in that it is characterized by a negative interpretation or evaluation of the positive situation.

In these studies, we wanted to investigate whether dampening also influences the impact of positive memory recall. From research within social psychology, it has been suggested that adopting an abstract/verbal processing mode for a positive memory (without an explicit focus on comparative thinking) can have enhancing effects (24). This may be because abstract processing can lead to positive elaborations or positive self-attributions (e.g., “I am a happy person,” “I am clever”) instead of interpreting the event as an isolated moment (24). However, it is plausible that if people characterized by a dampening response style think about the meaning of a positive memory, that they will less easily generate a positive meaning or may even reduce the positivity of the event. In this sense, dampening could be a potential moderator of the effect of an abstract/verbal thinking style on affect. This is akin to the idea that the impact of processing style on negative mood is conditional on individual differences in trait rumination [e.g., Ref. (25)].

In two studies, we investigated which thought processes determine the impact of positive memory recall on positive affect. In a first study, affective responding to concrete/imagery and abstract/verbal processing of a self-generated positive memory was investigated with a focus on dampening (and depressive symptoms) as moderator. In a second study, we also examined a comparative/verbal processing style.

## Study 1

We compared the affective impact of a concrete/imagery processing style focused on the sensorial and situation-specific details with an abstract/verbal processing defined as thinking about the broader meaning, causes, and consequences of an event. In addition, we investigated the moderating role of dampening.

In line with research on positive scenarios and positive memories [e.g., Ref. (5, 16)], we predicted that the processing of a positive memory in a concrete/imagery-based way would lead to increased positive affect compared to a verbal/abstract condition. With regard to an abstract/verbal processing style, we further predicted that this (immediate) change in positive affect would be smaller with increasing levels of dampening thoughts in daily life (due to minimizing or downgrading the positivity). Moreover, we did not only examine positive affect immediately after the processing of the memory but we also examined the affective impact of the processing styles after a short delay. We did this for two main reasons. First, we wanted to examine whether the processing styles would elicit a differential affective pattern after a delay. Second, we wanted to investigate the possibility that individuals characterized by higher levels of a dampening response style may still profit from positive memory recall, but only immediately. That is, it is possible that dampeners reveal an adequate initial affective response, but that the experienced positive feelings will be countered by downgrading thoughts after a delay. We predicted that these dampening thoughts would especially occur in the abstract/verbal condition, leading to a more negative affective change with increasing levels of dampening.

The study is conducted in a non-clinical sample. Given that reduced responding to positive material has been linked to depression, we also took the level of depressive symptomatology into account. Based on previous findings showing that mood improved after positive memory recall in never-depressed individuals but not in depressed individuals (11, 20), we predicted that increasing levels of depressive symptoms would be associated with worse affective outcomes.

## Method

### Participants

Seventy psychology students from the KU Leuven (University of Leuven) participated; see Table 1 for characteristics. Written informed consent was obtained at the beginning of the study. There was no compensation for participation.

**Table 1:** **Sample characteristics, memory characteristics (VAS), and affect (VAS) per condition (Study 1)**.

	Concrete/imagery condition *n* = 36		Abstract/verbal condition *n* = 34	
	Mean	SD	Mean	SD
Gender (freq female)	32		31	
Age	21.50	0.85	21.56	0.66
BDI-II	6.36	5.08	5.71	7.09
RPA-Dampening	10.81	2.65	11.50	3.39
Positivity of the event	8.52	1.33	8.56	1.06
Emotional (positive) intensity – past	8.41	1.29	8.44	1.43
Emotional (positive) intensity – retrieval	6.96	1.61	7.02	1.82
Positive affect
At baseline	6.18	2.03	6.41	2.16
After the induction	7.39	1.64	6.76	2.33
End of experiment	6.75	1.89	6.29	2.37

*BDI-II, Beck Depression Inventory-II; RPA-Dampening, the Dampening subscale of the Responses to Positive Affect questionnaire*.

### Measures

#### Affect assessment

Positive and negative affect were assessed using two visual analog scales, on which participants had to indicate how they felt at the moment. On the first scale, scores ranged from *totally not in a positive mood* (0) to *in a very positive mood* (100). The second scale ranged from *totally not dejected, “down,” sad, depressed* (0) to *very dejected, down, sad, depressed* (100)^1^.

#### Responses to positive affect questionnaire

The RPA (21) consists of 17 items scored on a 1 (*almost never*) to 4 (*almost always*) scale. Participants are requested to indicate how often they respond in a certain way when feeling happy, excited, or enthused. The RPA has three subscales: dampening (e.g., “My streak of luck is going to end soon”), self-focused positive rumination (e.g., “I am achieving everything”), and emotion-focused positive rumination (e.g., “Think about how happy you feel”). We used the 16-item Dutch version for which adequate psychometric properties are reported (22). In the current study, we focus on the 7-item dampening subscale, which had a Cronbach’s alpha of 0.72.

#### Beck depression inventory – second edition

The Beck depression inventory – second edition (BDI-II) (26) measures severity of depressive symptoms and consists of 21 four-choice statements. Participants are asked to indicate which of the four statements best describes how they felt during the past 2 weeks. The total BDI-II score is computed by summing the 21 scores (ranging from 0 to 3), which offers a total score ranging from 0 to 63, with higher scores indicating more depressive symptoms. We used the Dutch translation by Van der Does (27). Cronbach’s alpha in the present sample was 0.89.

#### Ruminative response scale (RRS) and the autobiographical memory test – minimal instructions

The ruminative response scale (RRS) (28) assesses the tendency to ruminate when feeling sad, down, or depressed. In the Autobiographical Memory Test (AMT)^2^ (29, 30), participants are given 1 min to write down a personal memory in response to 10 cue-words. The study was set up for a broader purpose than the one presented in the current paper. Consequently, the RRS and AMT are not reported in the Section “Results.”

#### Memory assessment

Three visual analog scales ranging from 0 to 100 were presented to assess the characteristics of the chosen event: (1) participants indicated that how positive they considered the event on a scale ranging from *totally not positive* to *very positive (the most positive emotional event from your life)*; (2) they rated the positive emotional intensity of the event at the moment it originally occurred on a scale ranging from *totally not emotional* to *very emotional*; (3) participants rated the positive emotional intensity that the episode had at the moment of recollection, from *totally not emotional* to *very emotional*.

#### Manipulation checks

Before and after the induction, level of self-focus and the focus on words or images were assessed using two visual analog scales ranging from 0 to 100: (1) at this moment, my attention is *totally not focused on myself* to *very much focused on myself*; (2) at this moment the things going through my head are *mainly words* to *mainly images*.

### Procedure

The study was presented as a study on the emotional life of university students and was conducted in a group setting. Participants were randomly allocated to one of the two conditions (abstract/verbal or concrete/imagery) via the distribution of different booklets. After giving their informed consent, participants completed a first version of the AMT. Next, participants completed the visual analog scales for negative mood, positive mood, self-focus, and focus on words or images. Participants were instructed to select a specific positive event from their own life, that is a life circumstance in which they had felt very happy and in a very positive mood. They were asked to write down a brief description of the specific episode. Next, they rated the chosen event on several characteristics (i.e., positivity, emotional intensity). This was followed by the processing style induction. In both conditions (abstract/verbal and concrete/imagery), participants were first instructed to think in the condition-specific style about their memory. Further, the processing style manipulations involved asking five condition-specific questions, on which participants were required to focus for a duration of 1 min per question.

In the *abstract/verbal condition*, participants were given the following instructions: *Try to understand the event you described and think about the possible causes of the event, the meanings and implications of the event for you and its consequences for you. Think in words about why the episode happened as it did, about the meanings and implications of the episode for you, and the consequences of the episode for you. Consider what caused the episode and judge what it says about you. Think about the episode in words and meanings, using verbal language of the sort that you use when you speak*. Then, they were asked to answer the following five questions: “W*hy did this event happen to you?; What was the meaning of this event in your life?; What were the consequences and implications of this event for you?; What did you think of yourself after the event?; What does this event say about your personality?.”* In the *concrete/imagery condition*, participants were given the following instructions: *Try to build up a detailed image of the event, as if you were reliving the event and as if a movie of the event was unfolding in your head. Spend a few moments imagining the event as if you were really there in the situation*. Then, they were asked to answer the following questions: “*What could you see around you?; What could you hear?; What could you feel, touch and experience in that situation?; What was happening around you?; What happened immediately after the event?.”* The instructions and questions were framed to parallel aspects of those used by Moberly and Watkins [(13), “concrete vs. abstract”] and Holmes and Mathews [(12), “ imagery vs. verbal”] as the imagery instructions appear virtually identical.

Immediately after the induction, students again completed the visual analog scales for negative mood, positive mood, self-focus, and focus on words or images. The delayed assessment of negative and positive mood was conducted after the completion of a second AMT, RRS, RPA, and BDI-II.

## Results

### Descriptive statistics and group comparisons

There were no differences between the two experimental conditions for any of the background variables (Table 1): BDI-II, *t*(68) = 0.45, *p* = 0.66, *d* = 0.11; RPA-Dampening, *t*(68) = 0.96, *p* = 0.34, *d* = 0.23; positive affect pre-induction, *t*(68) = 0.46, *p* = 0.65, *d* = 0.11; Distribution of gender was not significantly different between the two conditions, χ^2^(1) = 0.10, *p* = 0.75.

### Memory characteristics and content

To exclude the possibility that condition-specific effects on affect were due to baseline differences in memory characteristics, baseline ratings of the positive memory were compared between the two conditions. There were no significant differences between conditions on the positiveness of the event, *t*(68) = 0.13, *p* = 0.90, *d* = 0.03; emotional (positive) intensity of the event when the event happened, *t*(68) = 0.11, *p* = 0.91, *d* = 0.03; and the emotional (positive) intensity of the event at the moment of retrieval, *t*(68) = 0.15, *p* = 0.89, *d* = 0.03. Means and standard deviations are depicted in Table 1.

With regard to the content of the memories, we categorized the memories. Forty-three participants retrieved a *social* memory, primarily related to situations with family, friends, and partners (e.g., a nice evening out with friends, a special chat, being informed about a newborn; 11 of them were related to a romantic relationship). Other memories referred to an *achievement*: a scholarly (*n* = 22) or other achievement (*n* = 2). One participant reported a memory related to the enjoyment of *nature*, and two memories could not be categorized into one of the previous categories.

### Manipulation checks

#### Self-focus

To check that self-focus did not differ as a function of condition, a repeated measures ANOVA was conducted with time (before vs. after the processing style induction) as a within-subjects factor, condition (concrete/imagery vs. abstract/verbal) as a between-subjects factor, and self-focus as the dependent variable. The interaction between time and condition was not significant, *F*(1, 68) = 1.38, *p* = 0.24, $\eta_{p}^{2}=0.02.$ The main effect of time was significant, *F*(1, 68) = 6.47, *p* = 0.01, $\eta_{p}^{2}=0.09,$ *d* = 0.31, reflecting an increase in self-focus following the induction.

#### Processing style – imagery/verbal

A repeated measures ANOVA was conducted with time (before vs. after the processing style induction) as a within-subjects factor, condition (concrete/imagery vs. abstract/verbal) as a between-subjects factor, and focus on words vs. focus on images as the dependent variable. There was a main effect of time, *F*(1, 68) = 17.71, *p* < 0.001, $\eta_{p}^{2}=0.21.$ Critically, the interaction between time and condition was significant, *F*(1, 68) = 13.35, *p* < 0.001, $\eta_{p}^{2}=0.16,$ which is evidence for successful inductions. There was an increase (i.e., more use of images) in the concrete/imagery condition, *t*(35) = 5.97, *p* < 0.001, *d* = 0.99, but no change on the verbal/imagery scale in the abstract/verbal condition, *t*(33) = 0.37, *p* = 0.72, *d* = 0.06.

#### Processing style – concrete/abstract

Participants’ answers were coded on the level of abstract thinking (focus on causes, meanings, and implications) and concrete thinking (focus on concrete objects, sensory details, feelings, and sensations). Each answer was rated on a scale ranging from 1 (*not at all*) to 4 (*very much*). A mean concrete and abstract score was calculated for each participant. Distribution of these ratings did not allow parametrical test due to skewed distributions. A non-parametric median test indicated that the induction was successful. The median level of abstractness was significantly higher in the abstract/verbal condition (*Median* = 3.80) than in the concrete/imagery condition (*Median* = 1.00), χ^2^(1) = 62.28, *p* < 0.001. Further, the median level of concreteness in the concrete/imagery condition (*Median* = 3.20) was significantly higher than in the abstract/verbal condition (*Median* = 1.10), χ^2^(1) = 48.10, *p* < 0.001.

### The impact of processing style, depressive symptoms, and dampening on positive affect

To test if processing style (condition) influenced positive affect, and to test if RPA-Dampening or BDI-II acted as moderators of the association between processing style and positive affect, a hierarchical multiple regression analysis was conducted with change in positive affect from pre- to post-induction (positive affect after the induction minus positive affect at baseline) as dependent variable. Centered scores were used for RPA-Dampening and BDI-II. In the first step, condition, RPA-Dampening, and BDI-II were entered. In the second step, the interaction between condition and RPA-Dampening, and the interaction between condition and BDI-II were entered. Results are depicted in Table 2.

**Table 2:** **Hierarchical regression analyses: prediction of affective change (Study 1)**.

	DV: PA2PA1			DV: PA3PA2		
	*B*	SE*B*	β	*B*	SE*B*	β
Step 1
Condition	0.73*	**0.33**	**0.48**	−0.03	0.27	−0.03
RPA-Damp	−0.10	0.06	−0.20	0.07	0.05	0.17
BDI-II	0.10***	**0.03**	**0.39**	−0.08**	**0.02**	**−0.40**
Step 2
Condition	0.73*	**0.33**	**0.48**	−0.02	0.26	−0.02
RPA-Damp	−0.13	0.07	−0.27	−0.02	0.06	−0.05
BDI-II	0.08*	**0.04**	**0.33**	−0.07*	**0.03**	**−0.35**
Condition × RPA-Damp	0.09	0.12	0.17	0.23*	**0.09**	**0.58**
Condition × BDI-II	0.04	0.06	0.18	−0.02	0.05	−0.12

*PA2PA1 as criterion: R^2^ = 0.23 for Step 1, ΔR^2^ = 0.02 for Step 2 (p = 0.47); PA3PA2 as criterion: R^2^ = 0.15 for Step 1, ΔR^2^ = 0.07 for Step 2 (p = 0.06)*.

*DV, dependent variable. PA2PA1, positive affect after the induction minus positive affect at baseline; PA3PA2, positive affect at the end of the study minus positive affect after the induction; BDI-II, Beck Depression Inventory-II; RPA-Damp, Dampening scale of the Responses to Positive Affect questionnaire (RPA). Centered scores were used. Condition, abstract condition coded as 0 and concrete condition coded as 1. Significant predictors are indicated in bold. β’s indicate the regression coefficients when predictors (except condition) and the dependent variable were standardized*.

*p < 0.05.

**p < 0.01.

***p < 0.001.

Both models were significant, *p*s < 0.003. The interaction terms were both non-significant (*p*s > 0.45). To facilitate interpretation of the main effects, the first model without interaction terms was examined (*R*^2^ = 0.23). Importantly, there was a main effect of condition, *B* = 0.73, *p* = 0.03. The change in positive affect was higher in the concrete/imagery than in the abstract/verbal condition.

To investigate the direction of affective change within each condition, two paired-samples *t-*tests were conducted. Within the concrete/imagery condition, as predicted, positive affect increased significantly over time, *t*(35) = 4.77, *p* < 0.001, *d* = 0.79. Positive affect did not significantly change after abstract/verbal processing, *t*(33) = 1.52, *p* = 0.14, *d* = 0.26. RPA-Dampening was a non-significant predictor, *B* = −0.10, *p* = 0.08. There was a main effect of BDI-II, *B* = 0.10, *p* < 0.001, such that, somewhat unexpected, higher levels of depressive symptoms were associated with higher values of affective change. So the change in positive affect from pre- to post-induction was higher in the concrete/imagery than in the abstract/verbal condition.

To investigate whether there would also be a delayed effect of processing style, the hierarchical regression analysis was repeated with the change in positive affect from post-induction to the end of the experiment as dependent variable (*n* = 69, one missing value for the last affect rating), see Table 2. Both models were significant, *p*s < 0.02. In the first step, *R*^2^ = 0.15, there was only a main effect of BDI-II, *B* = −0.08, *p* = 0.001, which was in the opposite direction than the main effect of BDI-II in the previous analysis (i.e., the immediate change in positive affect). The change in positive affect did not significantly differ between conditions. To investigate the direction of affective change during the delay, a paired-samples *t-*test was conducted. Positive affect decreased significantly during the delay, *t*(68) = 3.77, *p* < 0.001, *d* = 0.45, meaning that, the increased level of positive affect in the concrete/imagery condition was not fully maintained after a delay. However, positive affect at the end of the experiment was still significantly higher than at baseline in the concrete/imagery condition, *t*(34) = 2.20, *p* = 0.04, *d* = 0.37. Although positive affect slightly decreased during the delay, there was no difference between positive affect at baseline and at the end of the experiment in the abstract/verbal condition, *t*(33) = 0.65, *p* = 0.52, *d* = 0.11.

The inclusion of the interaction terms in step two added marginally significantly to the explained variance, Δ*R*^2^ = 0.07, *p* = 0.059. Importantly, this second step consisted of a non-significant interaction between BDI-II and condition, whereas the interaction between condition and RPA-Dampening was significant, *B* = 0.23, *p* = 0.02. The interaction with RPA-Dampening revealed a significant positive relation between change in positive affect and RPA-Dampening in the concrete/imagery condition, *B* = 0.21, β = 0.53, *p* = 0.01, and not in the abstract/verbal condition, *p* = 0.72. This means that the higher trait dampening in the concrete condition, the smaller the decrease in positive affect during the delay.

## Discussion

The affective impact of memory recall is not only determined by the valence of the event but also by how it is processed. The current study aimed to investigate the impact of concrete/imagery vs. abstract/verbal processing of recall of a positive memory on positive affect in a non-clinical sample. In addition, the role of dampening and depressive symptoms as potential moderators was investigated. As predicted, we found that positive memory recall in a concrete/imagery way increased positive affect. This is in line with earlier studies on imagining standardized positive scenarios [e.g., Ref. (5, 15)] and with positive memory recall to repair sad mood (16). Abstract/verbal processing did not influence positive affect in our study. This is, as a general outcome here for positive autobiographical memories, less “negative” than an actual mood deterioration (reduction in positive affect) found after verbally/abstractly processing hypothetical positive situations (5, 15). This suggests that abstract/verbal processing of a positive information may not always lead to a mood deterioration [see also Ref. (5, 20, 24)], though it does not appear to boost positive mood.

The affective impact of abstract/verbal processing was in our sample not related to people’s tendency to dampen positive affect in daily life. This suggests that, the extent to which people use dampening thoughts in response to positivity (as measured via the RPA) may not influence the immediate affective impact of positive memory recall. However, the affective response to positive memory recall was higher for people with higher levels of depressive symptoms. To clarify this main effect of BDI-II, we present a group approach. In the “high BDI-II group” (=BDI-II above median), positive affect significantly increased from baseline to post-induction (*p* < 0.001, *d* = 0.81), but there was no significant change in affect in the “low-BDI-II group” (*p* = 0.13, *d* = 0.26). This means that “dysphoric” participants were more responsive to positive memory recall. This was not expected based on research that revealed a reduced responding to non-autobiographical positive pictures in people with depressive disorder [e.g., Ref. (3)] and a worsening of sad mood after positive memory recall (11). An explanation could be that “dysphoric” participants retrieved a more positive memory. However, positiveness of the event was unrelated to BDI-II, *r* = 0.01. The counterintuitive main effect of depressive symptoms might also be caused by a lower baseline state affect in dysphoric participants, and so there was more room for affective improvement. Baseline positive affect was indeed lower in the “high BDI-II group” compared to the “low BDI-II group,” *p* = 0.048. The main effect of BDI-II that we observed, however, is in line with the so-called *mood-brightening effect* observed in naturalistic settings: several studies have shown that in depressed individuals and in individuals with elevated depressive symptomatology, positive events are followed by a greater decrease in negative affect (31) and a greater increase in positive affect (32) than in controls. Further research is needed to sort out the nature of this effect.

We were also interested in the delayed affective responding to memory recall. Overall, following a short delay, positive affect decreased, but still remained slightly higher than at baseline for the imagery/concrete condition. We make two notes. First, the affective change was worse for people with increasing levels of depressive symptoms. Again, to clarify this by a group approach, after the delay, the “high BDI-II” group experienced a drop in positive affect, *p* < 0.001, *d* = 0.79, while this was not the case for the “low-BDI-II group,” *p* = 0.38, *d* = 0.15. This means that the increased immediate responding in people with more depressive symptoms had the downside of a greater drop after a short interval. Depressed individuals fear or suppress positive affect (33), possibly because they want to prevent that they will experience such a drop in positive affect or that they will even feel worse afterwards. Second, on top of the main effect of BDI-II, concrete/imagery processing had a positive impact on the delayed affective response in participants with increasing levels of a dampening response style. The results suggest that after concrete memory recall, positive affect is better maintained in individuals who dampen more, which points to the importance of processing positive events in a concrete manner for “vulnerable” individuals (in this case, high trait dampeners). One possible explanation for this result is that the “dampeners” were distracted from their habitual “abstract” (dampening) responses.

Some remarks can be made about this study. First, there were no restrictions on the content of the chosen memory. Consequently, participants retrieved different types of memories, for example social or interpersonal (e.g., a nice chat with friends) or achievements (e.g., good exam results). It is plausible that meanings and consequences for social memories are generally different than for scholarly achievements. Second, there was no assessment of the temporal distance of the memory. Given that age can influence phenomenal characteristics of past events (34), it would be better to check for potential differences between conditions on memory age. Third, and critical to the next study, it has been suggested that making (unfavorable) comparisons between positive scenarios and the (less fortunate) self (5) or between a past happy event and the (less positive) current life/self might be a crucial process that undermines the benefits in positive information recall [e.g., Ref. (16)]. Therefore, an interesting next step entails the comparison of three processing styles in their affective outcome on positive memories: (1) a concrete/imagery style focused on the sensorial and situation-specific details, vs. (2) abstract/verbal processing defined as thinking about the broader meaning, causes, and consequences, vs. (3) an abstract/verbal thinking style focused on making comparisons between the current self/life and the past self/life (“comparative/verbal”). The comparison with a comparative/verbal thinking style would show whether it is a non-concrete/verbal processing style that is unbeneficial or whether it is specifically making (unfavorable) comparisons that is afforded by this processing style, which undermines positive memory recall [cf. (5)].

## Study 2

A second study was designed with three objectives: (1) to address limitations of the first study, (2) to examine a third way in which a positive memory could be processed by making verbal comparisons, and (3) to examine mental imagery perspective.

Concerning the first objective, the content variability of the memories in Study 1 was addressed by using the same memory for all participants. Passing an entrance exam is a necessary condition to study medicine or dentistry in Flanders. As a consequence, students of medicine and dentistry share this achievement. This guided retrieval also aided to control the time period of the memory (i.e., restricted to July, August, and September).

With regard to the second objective, a concrete/imagery-based processing style was generally better than an abstract/verbal processing style in Study 1. However, an abstract/verbal processing style did not significantly deteriorate affect, which was the case in some other studies with positive hypothetical scenarios [e.g., Ref. (5)]. It is hypothesized that it is especially making unfavorable comparisons with positive events that is detrimental [e.g., Ref. (5), cf. (35)]. Extending this to positive memories, past events might act as a point of comparison to evaluate one’s present life (16, 36). Therefore, pleasant events could contrast with someone’s less satisfactory current life, emphasizing negative aspects of the current situation or self-deterioration. Joormann and Siemer (37) and Joormann et al. (11) suggested that for depressed individuals a positive memory may highlight that one is not that positive anymore. Although the memory itself is positive, it comes to underline the discrepancy between positive times in one’s past and the sad mood or the less favorable situation now [see also Ref. (16)].

We included a third processing style in which individuals compare their current self (current life) with the person they were (the life they had) at the time the positive memory was investigated (comparative/verbal). It has been suggested that especially for depressed individuals a positive memory highlights the discrepancy between the happy past moment and a sad current life, or negative current self [e.g., Ref. (11)]. We predicted that after adopting such a comparative/verbal thinking style, change in positive affect would be less beneficial, especially with increasing levels of depressive symptoms. We had no specific predictions with regard to the delayed responding. For the concrete/imagery and abstract/verbal styles, predictions are the same as in Study 1.

A third objective was to investigate the effect of processing style on imagery perspective. Episodic memory is defined by including sensory perceptual details (38, 39). That is, when remembering a specific event, people typically generate mental images. These mental images can be experienced from an observer perspective, in which people observe themselves from the outside, or from a field perspective, in which people experience the event again through their own eyes (40). What was our reasoning behind this third, perspective-related study objective? For example, research showed that hypothetical situations, which are described at an abstract level (e.g., the broader goal of the action), are more likely to be imagined from an observer perspective than concretely described actions (41). Similar results are found if participants think about the broader meaning of a personal past event (graduation) vs. if they focus on the experience (concrete details) (42). In addition, some studies indicate that an observer perspective entails a negative evaluative component. That is, seeing oneself from the outside facilitates a negative evaluation of the self and the imagined situation, or facilitates unfavorable comparisons between the self and a positive situation (43–46) and could contribute to a negative response in the face of recalling a positive event. Bringing this together, we might expect less field (and more observer) perspective after abstract/verbal processing (as compared to concrete processing). And, observer imagery in the abstract/verbal condition could be more pronounced with increasing levels of dampening (as dampening could be regarded as negatively evaluating something positive). We also expected less field (and more observer) perspective after comparative/verbal thinking [cf. Ref. (43)].

## Method

### Participants

Participants were students of medicine and dentistry from the KU Leuven (University of Leuven). They were invited through an online university platform to participate in an online study. Participants who completed all measures were included (*n* = 159). Most participants were students of medicine (*n* = 123), 35 participants studied dentistry, and 1 participant combined both studies (see Table 3 for characteristics). Twenty randomly selected participants received a gift voucher in exchange for participation. The study was approved by the Ethical Committee of the Faculty of Psychology and Educational Sciences, KU Leuven (University of Leuven). Informed consent was obtained online before the start of the study.

**Table 3:** **Sample characteristics, memory characteristics, and affect (Study 2)**.

	Concrete/imagery condition *n* = 53		Abstract/verbal condition *n* = 53		Comparative/verbal condition *n* = 53	
	Mean	SD	Mean	SD	Mean	SD
Gender (freq female)	35		31		38	
Age	21.15	2.46	20.38	1.44	20.83	4.82
DASS-Depression	4.09	3.43	4.57	3.75	3.83	5.57
RPA-Dampening	12.47	3.16	13.38	4.28	12.47	3.78
Number of sittings	2.02	1.22	1.96	1.02	2.08	1.02
Positivity of the event	7.77	0.99	8.08	0.81	7.57	1.01
Important/meaningful memory	7.66	1.87	7.77	1.75	7.34	1.69
Concentration	8.04	1.27	8.13	0.88	7.91	1.29
Positive affect
Baseline	5.92	1.58	6.11	1.38	6.51	1.01
After the induction	6.62	1.68	6.64	1.27	6.51	1.49
End of survey	5.92	1.65	5.96	1.49	6.17	1.67
Post-induction imagery perspective
Field	4.96	1.66	4.70	1.62	4.77	1.55
Observer	3.66	1.78	3.96	1.61	3.72	1.81

*DASS-Depression, The Depression subscale of the Depression Anxiety and Stress Scale; RPA-Dampening, The Dampening subscale of the Responses to Positive Affect questionnaire. Number of sittings, how many times participants attended the entrance exam*.

### Measures

#### Affect assessment

Administration of positive and negative affect was as in Study 1. However, this time, participants responded using a 9-point Likert scale (cf. internet-based nature of Study 2).

#### Responses to positive affect questionnaire – dampening subscale

See Study 1 (21). The 7-item Dampening subscale was administered, with a Cronbach’s alpha of 0.78.

#### Depression anxiety stress scale – depression subscale

The Depression anxiety stress scale – depression subscale (DASS-D) (47) assesses the severity of depressive symptoms for the past 7 days. Item scores range from 0 (*did not apply to me at all*) to 3 (*applied to me very much or most of the time*). The basic DASS-D contains 14 items; we used the short version of the DASS-D with 7 items. The Dutch version by de Beurs et al. (48) was used. Cronbach’s alpha in the present sample was 0.84. We did not use the 21-item BDI-II as in Study 1 to limit the time of the online assessment.

#### Memory characteristics

Several Likert scales were used to assess baseline characteristics of the event or memory, and to assess memory characteristics after the induction. At baseline, participants were asked how *positive* they found the moment when they were informed that they passed the entrance exam (cf. Study 1, but, again not rated on a visual analog scale but using a 9-point Likert scale). Participants additionally rated to what extent the memory was an important and meaningful memory for them personally, using a scale ranging from *totally not important and meaningful* to *very much important and meaningful*. Post-induction, students rated to what extent passing the entrance exam was characteristic for the person they are now (this scale will not be discussed in the current paper). Age of the event was assessed by determining the moment (month and year) the event took place. Finally, we assessed imagery perspective. For that, participants were first asked to make an image of the moment they were informed that they passed the entrance exam. Next, they completed two 7-point scales: the first scale ranged from (1) *totally not first person* to (7) *completely first person*; the second scale ranged from (1) *totally not third person* to (7) *completely third person* [similar scales were used in Ref. (49)].

#### Concentration

Because of the online format, participants were asked to what extent they were able to complete the online tasks with concentration (without being distracted) on a 9-point scale ranging from *totally not* (1) to *very good* (9). Although participants are randomized to condition, we wanted to check whether individuals from the three conditions would differ on how focused they were while completing the questions.

### Procedure

After completing the online informed consent, participants filled out a first measure of affect and self-focus (on a 9-point Likert scale). Next, they were asked to rate the positivity of a specific event, namely “the moment you were informed that you passed the entrance exam.” This was followed by a rating of how important and meaningful the memory is. Next, participants were randomized to one of the three conditions. Randomization to condition was conducted automatically via the survey software Qualtrics. Processing style was manipulated using a general condition-specific instruction followed by seven condition-specific questions. Participants had to type their answer to each question in full sentences. The induction was based on, and adapted from previous research (5, 12, 13, 15, 16, 20, 50). Unlike in Study 1, there was no time restriction per question used in the online assessment.

In the *concrete/imagery condition*, participants were prompted to focus on the moment, to replay the event in their mind as a movie, and to form a sensory mental image. Comparable to Study 1, the condition-specific questions were what they could hear, see, and what they felt when receiving their exam result, and what they did immediately after. In addition, we prompted for more details by asking how the moment unfolded, how they received the exam result (e.g., via e-mail, phone), where they were, and how bystanders behaved.

In the *abstract/verbal condition*, participants were asked to verbally generate meanings about the situation. Comparable to Study 1, they were asked what the consequences and implications were, how they thought about themselves, what the meaning was of the event for their life, and what the performance said about their capabilities. Instead of asking why the event happened to them, they were asked more specifically how they explained that they passed (succeeded in) the exam. Two extra questions assessed *why* they felt the way they did, and whether the result (performance) was as expected.

In the *comparative/verbal condition*, again, participants were asked to use verbal language. They were asked how the person they are now differs from the person they were at that moment, and how their life is different now. They were also asked how they expected their life would unfold at the time of the memory vs. how it has turned out. One question focused on a social comparison (how their performance compared to that of other students who participated in the same exam).

After the processing induction, all participants completed a second measure of affect and self-focus, followed by the assessment of how characteristic the memory is and imagery perspective. Next, we asked for demographics (such as gender and study level) and details about the entrance exam (such as time). The Dampening subscale of the RPA and the Depression scale of the DASS were completed and the study ended with a final measure of affect and an assessment of their concentration during the online study.

## Results

### Descriptive statistics and group comparisons

Five one-way ANOVAs showed that the conditions did not significantly differ on age, depression score (DASS-D), level of dampening (RPA-Dampening), concentration, or baseline positive affect, 0.08 < *p*s < 0.60. There were also no gender differences, χ^2^(2) = 2.06, *p* = 0.36 (for descriptive, see Table 3).

### Memory characteristics

To exclude that the effects on affect were due to baseline memory differences, several one-way ANOVAs were conducted. Conditions did not differ on the mean number of times participants sat the entrance exam, *F*(2, 156) = 0.14, *p* = 0.87, $\eta_{p}^{2}=0.002.$ or the level of importance/meaningfulness of the memory, *F*(2, 156) = 0.86, *p* = 0.43, $\eta_{p}^{2}=0.01.$ As expected, the mean positivity rate was high and no one rated the entrance exam as totally not positive. However, conditions significantly differed on how positive they perceived passing the entrance exam, *F*(2, 156) = 3.93, *p* = 0.02, $\eta_{p}^{2}=0.05.$ Notice that this difference cannot be driven by the processing induction as that was afterward. *Post hoc t*-tests revealed that positivity was significantly higher in the abstract/verbal compared to the comparative/verbal condition, *t*(104) = 2.87, *p* = 0.005, *d* = 0.56. Therefore, we controlled for positivity in the following regression analyses. The entrance exam took place during the year of the current research to a maximum of 4 years previously, with the exception of one participant for whom it was 6 years ago. Conditions did not differ for the age of the event, *F*(2, 156) = 1.16, *p* = 0.32, $\eta_{p}^{2}=0.02.$ Means and SDs are reported in Table 3.

### Manipulation checks

#### Self-focus

To verify if self-focus did not differ as a function of condition, a repeated measures ANOVA was conducted with time (before vs. after the processing style induction) as a within-subjects factor, condition (concrete/imagery, abstract/verbal, comparative/verbal) as a between-subjects factor, and self-focus as the dependent variable. The interaction between time and condition was not significant, *F*(2, 156) = 0.38, *p* = 0.69, $\eta_{p}^{2}=0.005.$ The main effect of time was significant, *F*(1, 156) = 49.05, *p* < 0.001, $\eta_{p}^{2}=0.24,$ *d* = 0.56. Comparable to Study 1, self-focus increased after the processing style inductions.

#### Processing style

To analyze responses to the questions, 21 participants per condition were randomly selected. This group (*n* = 63) was comparable to the others in terms of baseline memory characteristics and DASS-D, *p*s > 0.10, but despite the random selection, they scored lower on RPA-Dampening, *p* = 0.01. The written induction was coded in terms of level of *concreteness* (to what extent does the answer focus on sensorial information or on the concrete unfolding of the situation), *abstractness* (to what extent does the answer tell you something about meanings, causes (why they passed the exam), consequences, or why they felt the way they did), and the level of *comparative thinking* (to what extent is the answer about the person at the time of the event and the comparison with then vs. now or vs. others, the expectations then vs. how it is now, and the time in between).

We used a scale ranging from 1 (*not at all*) to 5 (*very much*). Distribution of the ratings did not allow significance tests (limited range on the scores within each condition), therefore descriptives are reported. As expected, comparative thinking was high in the comparative condition (*M* = 4.05, range 3–5), whereas there was almost no comparative thinking in the other two conditions (range 1–2). Ratings for concrete thinking were high in the concrete/imagery condition (*M* = 4.86, range 4–5), but less pronounced in the other two conditions (range 1–3). Abstract thinking was high in the abstract/verbal condition (*M* = 4.67, range 4–5), low in the concrete/imagery condition (*M* = 1.38), and somewhat represented in the comparative condition (*M* = 2.90, range 1–4). In each condition, 11 of the 21 texts were randomly selected and recoded by a second researcher. For each scale, an intraclass correlation coefficient between the score of rater 1 and rater 2 was calculated. The correlation coefficients are 0.94 for concrete thinking, 0.85 for abstract thinking, and 0.95 for comparative, suggesting a good interrater reliability.

### The impact of processing style and depressive symptoms on positive affect

To test the impact of processing style on positive affect and to test the hypothesis that there would be an interaction of condition and depressive symptoms, we conducted a hierarchical multiple regression analysis with the change in positive affect from pre- to post-induction (positive affect after the induction minus positive affect at baseline) as the criterion variable. Because of the increased number of predictors in the second study, regression analyses were separately conducted for DASS-D and RPA-Dampening. In Step 1, positivity of the event was entered as a control variable (due to baseline differences between conditions). In Step 2, DASS-D scores, and two dummy variables to specify condition were included. The abstract/verbal condition functioned as the reference group. Finally, in Step 3, the interaction terms – which specify the interaction between condition and depressive symptoms – were entered. For DASS-D, and the positivity of the event, we used centered scores. Table 4 summarizes the hierarchical regression analysis. For all three steps, the model was significant, *p* < 0.001. In the first step, positivity of the event was a significant predictor of change in positive affect. Next, in Step 2, the level of depressive symptoms was not a significant predictor. There was a significant main effect of condition, in such that the change in positive affect was higher in the abstract/verbal compared to the comparative/verbal condition, but the abstract/verbal and concrete/imagery condition did not significantly differ.

**Table 4:** **Hierarchical regression analysis: prediction of affective change after processing of the positive memory (Study 2)**.

	*B*	SE*B*	β
Step 1
**Positivity**	0.29***	**0.09**	**0.27**
Step 2
**Positivity**	0.25**	**0.09**	**0.23**
Dummy 1	0.24	0.20	0.23
**Dummy 2**	−0.41*	**0.20**	**−0.39**
DASS-D	−0.01	0.02	−0.04
Step 3
**Positivity**	0.26**	**0.09**	**0.23**
Dummy 1	0.26	0.19	0.25
**Dummy 2**	−0.42*	**0.20**	**−0.39**
DASS-D	0.03	0.04	0.10
Dummy 1 × DASS	−0.001	0.06	−0.004
**Dummy 2 × DASS**	−0.12*	**0.05**	**−0.40**

*R^2^ = 0.07 for Step 1, ΔR^2^ = 0.06 for Step 2 (p = 0.01), ΔR^2^ = 0.03 for Step 3 (p = 0.046)*.

*PA2PA1, change in positive affect from pre- to post-induction (positive affect after the induction minus positive affect at baseline); Positivity, self-reported positivity of the event; DASS-D, Depression subscale of the Depression Anxiety Stress Scales. Centered scores were used. Dummy 1, concrete/imagery condition coded as 1, the other two conditions as 0; Dummy 2, comparative/verbal condition coded as 1, the other two conditions as 0. Significant predictors are indicated in bold. β indicates the regression coefficients when predictors (except condition) and the dependent variable were standardized*.

*p < 0.05.

**p < 0.01.

***p < 0.001.

To know in what direction affect changed from pre- to post-induction within each condition, we conducted three paired-samples *t*-tests. Means and SDs are depicted in Table 3. As predicted, in the concrete/imagery condition, positive affect significantly increased, *t*(52) = 5.45, *p* < 0.001, *d* = 0.75 (*M* = 0.70, SD = 0.93). In the abstract/verbal condition (for which the mean change did not significantly differ from the concrete/imagery condition), positive affect also significantly increased, *t*(52) = 3.73, *p* < 0.001, *d* = 0.51 (*M* = 0.53, SD = 1.03). In the comparative condition, positive affect remained unchanged after the induction, *t*(52) = 0, *d* = 0 (*M* = 0, SD = 1.09).

In the comparative/verbal condition, affective change was dependent on depressive symptoms. That is, importantly and as hypothesized, the main effect of condition was qualified by an interaction effect in the third step. The slope representing the relationship between DASS-D and affective change significantly differed between the abstract/verbal condition and the comparative/verbal condition. To interpret this interaction, we present the slope for the relation between DASS-D and affective change within each condition. As expected, in the comparative condition, higher DASS-D scores were associated with a lower value of the change score, *B* = −0.09, β = −0.30 *p* = 0.02, whereas the slope did not significantly differ from zero in the concrete/imagery condition, *B* = 0.03, β = 0.09, *p* = 0.51, and in the abstract/verbal condition (see Table 4).

### The impact of processing style and dampening on positive affect

The same hierarchical regression analysis as described above was conducted, however, with DASS-D score changed by RPA-Dampening. This regression analysis did, again, reveal the same main effect of condition. However, there was no main effect of RPA-Dampening, and the two interaction terms did not significantly add to the explained variance of change in positive affect (Δ*R*^2^ < 0.001).

### The delayed affective impact of processing style

The regression analyses were repeated with, as the criterion, the change in positive affect from post-induction to the end of the survey. The models with DASS-D as well as the models with RPA-Dampening were non-significant (*p*s > 0.14).

From post-induction to the end of the survey, positive affect significantly decreased in the concrete/imagery and the abstract/verbal condition (*p*s < 0.001), but not in the comparative condition (*p* = 0.08), such that in all three conditions, positive affect at the end of the survey did not differ from baseline affect, *p*s > 0.10.

### The impact of processing style on imagery perspective

Using two one-way ANOVAs, the effect of processing style (condition) on field perspective and observer perspective was examined (see Table 3). Conditions did not differentially influence imagery perspective, *p*s > 0.63. To inspect associations with RPA-Dampening and DASS-D, correlations were calculated within each condition. RPA-Dampening was not significantly associated with imagery perspective. With regard to the DASS-D, depressive symptoms were negatively associated with field perspective ratings in the comparative condition, *r*(53) = −0.28, *p* = 0.04, but not in the other two conditions, *r*s < −0.14, *p*s > 0.33. However, the correlation in the comparative condition did not significantly differ from the correlations in the other two conditions (*p*s > 0.41).

## Discussion

The aim of this study was to investigate the effect of processing style manipulation of retrieval of a positive memory on positive affect (immediate and delayed) and on mental imagery perspective. Based on previous investigations, we compared three thinking styles: concrete/imagery, abstract/verbal, and comparative/verbal. We also investigated the role of self-reported dampening during daily life and current depressive symptoms as potential moderators. All participants thought back about the same achievement – an exam.

As expected, positive affect increased after concrete/imagery processing of the positive memory, replicating our Study 1, and extending results in previous related papers. However, unlike Study 1, here the abstract/verbal condition did also lead to an increase in positive affect, showing that generally abstract/verbal thinking might not be maladaptive under certain conditions. This would at least be without a focus on the comparative thinking element [cf. (5)] and for a positive content. A necessary condition to gain positive mood while generally abstract/verbal might be that the positive memory receives a strong meaning in someone’s life: this was reflected in the generation of positive meanings in the abstract/verbal condition (e.g., “*it meant a lot for me,” “it teaches me that with dedication you can excel in life,” “it showed that I have more capacities than I thought”*).

In analogy with our results, an abstract processing style in combination with a lab-induced success experience yielded the same affective outcomes as a concrete processing style (51). This was the case for low as well as high dysphorics. Non-deleterious effects of abstract processing have also been shown in social psychology. Marigold et al. (24) hypothesized that people with low self-esteem (who tend to dampen the positivity of a partner’s compliment) would profit from an abstract processing of a partner’s compliment [see also Ref. (52)]. Indeed, people with low self-esteem who were prompted to verbally describe the meaning of a past compliment felt happier compared to participants who were asked to describe the isolated, concrete details of the moment they received a past compliment (24).

Unexpectedly, the immediate affective response as well as the delayed responding was independent of self-reported dampening responses during daily life, partially replicating results from Study 1. The fact that the affective response in the concrete/imagery and abstract/verbal processing conditions was independent of depressive symptoms suggests that people with higher levels of depressive symptoms are, at least under certain circumstances, able to profit from positive memory recall [e.g., when thinking back about important achievements without making (unfavorable) comparisons].

As expected, making comparisons between the current self/situation with the past self/situation in the memory was less helpful and did not increase positive mood over time. Importantly, in line with the prediction, the affective impact of making such comparisons was worse with increasing levels of depressive symptoms. It is unlikely that this is driven by a ceiling effect for individuals with higher levels of depressive symptoms, given that they started with lower positive affect at baseline. Plausibly, a general discrepancy between the current negative self or life situation and the past positive situation drives this effect. In this reasoning, we assume that especially in individuals with depressive symptoms a discrepancy between the past happy situations and the current life may exist, driving unfavorable comparisons. This discrepancy is less easily triggered while using a more concrete/imagery or abstract/verbal thinking style without explicit comparisons. The “risky” side of comparing oneself (or one’s current life circumstances) with a positive event was also present in Holmes et al. (5) for hypothetical events. Our study now reveals that this also pertains to real personal positive events from the past. An implication is that if working with positive stimuli, we should take care not to get stuck in unbeneficial comparative thinking.

Importantly, passing an entrance exam is particularly related to expectations and predictions about the future university years. Therefore, making comparisons between now and then might easily prompt unachieved expectancies with regard to one’s academic performances. Indeed, if we control for how satisfied participants are with how their studies unfold, the association between depressive symptoms and affect change in the comparative condition becomes marginally significant (β = −0.23, *p* = 0.085), although the interaction between condition and depressive symptoms remains significant. This suggests that the effect may also be driven by the current state of the life domain from the memory.

The three thinking styles did not differentially influence imagery perspective of the recalled event. However, in another between-subjects investigation, students who thought abstractly about their graduation were more likely to recall the memory from an observer perspective relative to students who adopted a concrete processing style (42). The induction was comparable but not identical, though, speculatively, the differential imagery measure between studies (dichotomous vs. continuous) might be a candidate explanation for the mixed results.

## General Discussion

In two studies, we investigated the impact of processing style during positive memory recall on positive affect. We also considered the role of dampening and depressive symptoms as potential moderators.

A focus on the sensory experience of the positive event (concrete/imagery processing) improved positive affect for a positive memory that was self-generated (Study 1) and experimenter-chosen (i.e., an academic achievement, Study 2). This parallels evidence with standardized positive hypothetical scenarios [e.g., Ref. (5, 15)] and extends research on the concrete recall of a positive memory to repair sad mood in (previously) depressed individuals (16) to increasing positive affect in the absence of a negative mood induction in a non-clinical sample.

Results also support the development of training procedures to help make depressed individuals more specific and concrete in the retrieval of their memories [e.g., Ref. (53)] and boost their positive mental imagery of hypothetical and future events [e.g., Ref. (9, 54–56)]. It is likely that the combination of the concrete/imagery processing style with a positive content drives the increase in positive affect [for a control with a neutral or negative content, see Ref. (57, 58)].

Considering abstract/verbal processing of a memory (i.e., thinking about the broader meaning, causes, and consequences of the event), in neither of the two studies did abstract/verbal processing cause significant mood deterioration; however, it failed to lead to a beneficial effect in the controlled lab environment in Study 1. This affective outcome is thus less detrimental than contrast effects (i.e., mood deterioration) with positive memories observed in a depressed sample (11) and with hypothetical positive events in non-clinical samples (5, 15, 43). Speculatively, the different outcome for abstract/verbal processing in Study 1 and Study 2 might be driven by the content of the memory or the control over the study environment. Another possibility is that the abstract instructions in the second study were adjusted to the memory and included positive connotations (e.g., what do you think about your *performance*). This might have stimulated to derive a positive meaning. Indeed, Marigold et al. (24) confirmed that such subtle positive framework avoids self-evaluative concerns in individuals with low self-esteem.

We also examined delayed responses to memory recall. Delayed responses were characterized by a decrease in positive affect in both studies. In general, the pattern indicated that people ended at their baseline level of positive affect (note that in the imagery/concrete condition of Study 1 positive affect did not entirely drop to baseline after the delay). In both studies, the delay phase included the assessment of questionnaires (such as a depressive symptom scale), which might have been mood triggering. Ideally, positive imagery protects against later mood triggers. In previous investigations, an elaborated imagery training protected against future negative mood (5) and positive affect was maintained during a 10 min neutral filler (17).

With regard to dampening as a suggested moderator, unexpectedly, the immediate as well as the delayed impact of abstract/verbal processing was not worse for people characterized by dampening responses. This means that although people have a stronger dampening cognitive style in response to positive affect, they do not appear more vulnerable to the effects of abstract/verbal thinking about a positive memory. We should note, however, that high levels of dampening might be not sufficiently resembled in the present samples. We also note that we investigated “trait” dampening responses to positive affect. For future research, it would be interesting to include a state measure of dampening [cf. LeMoult et al. (59) for state rumination].

In the second study, we compared two less concrete/imagery-based processing styles (abstract/verbal vs. comparative/verbal). As hypothesized, comparative/verbal thinking yielded different responding than abstract/verbal thinking. This suggests that verbal processing itself is not necessarily a problem, but rather it is the combination with making (unfavorable) comparisons, which makes it less adaptive. Importantly, comparative thinking was especially detrimental for individuals with higher levels of depressive symptoms. Overall, this suggests that, at least with this type of achievement memory, comparative thinking (presumably *unfavorable* comparisons) turns out to be the unbeneficial factor and not the abstractness of thinking as such. Interestingly, given that abstract/verbal processing did not increase positive affect in Study 1, but did in Study 2, it is unclear whether the difference between abstract and comparative-based thinking in Study 2 was solely driven by the negative ingredients of comparative thinking or also by positive aspects of the abstract/verbal induction. It will be important to replicate this study and investigate it with different types of memories.

The interpretation of the results and comparison of the two studies have some final limitations. First, we are aware that the comparison of the two studies is limited due to method differences. For example, two different measures of depressive symptoms were used and the numerical anchors of the affect scales differed. Second, both studies were limited to student samples and we did not collect diagnostic information on depressive status. Therefore, we should remain cautious when interpreting our results in the context of clinically depressed patients or persons at risk for (relapse/recurrence of) clinical depression. Third, in both studies, we did not include a neutral control condition, therefore we cannot conclude whether the differences between conditions are driven by the active positive effect of a concrete/imagery-based processing style. Fourth, both studies were not conducted in an individual setting. Although similar manipulations were conducted in a group context (50) or via internet (24), it is recommended to repeat the study in an individual setting.

Overall, the present studies support the potential of trainings in positive mental imagery with autobiographical material. In addition, the studies stress taking care of thought patterns when working with (autobiographical) positive stimuli in therapy and underline the need for research into parameters that determine the outcome of abstract/verbal processing.

## Conflict of Interest Statement

The authors declare that the research was conducted in the absence of any commercial or financial relationships that could be construed as a potential conflict of interest.

## Footnotes

^1^The anchors of all visual analog scale were 0 and 100. Unfortunately, due to photocopying, the scales were 101 instead of 100 mm. The answers were processed in centimeters and thus ranged from 0 to 10.1.

^2^During the AMT (written format), five positive and five negative cue words are presented in an alternating order. We used the “Minimal Instructions” version of the AMT (30). In this version, participants are asked to retrieve memories, without emphasizing in the instructions that these should be specific. At the end, participants were requested to code their memories for specificity and imagery perspective (dichotomous: first or third-person perspective). The administration of imagery perspective during the baseline AMT allowed us to investigate the relation between dampening and the use of an observer perspective during “unmanipulated” memory retrieval. For each subject, the number of observer and field memories was calculated. RPA-Dampening was positively correlated with the number of observer memories, *r*(68) = 0.37, *p* = 0.002 and negatively correlated with the number of field memories, *r*(68) = −0.32, *p* = 0.01 (*n* = 68, two persons without perspective ratings). These correlations had comparable effect sizes after control for BDI-II. The correlations were independent of the valence of the cue word.

## References

[1] Carl JR Soskin DP Kerns C Barlow DH. Positive emotion regulation in emotional disorders: a theoretical review. Clin Psychol Rev (2013) 33:343–60. 10.1016/j.cpr.2013.01.003 23399829

[2] Dunn BD. Helping depressed clients reconnect to positive emotion experience: current insights and future directions. Clin Psychol (2012) 19:326–40. 10.1002/cpp 22674611

[3] Dunn BD Dalgleish T Lawrence AD Cusack R Ogilvie AD. Categorical and dimensional reports of experienced affect to emotion-inducing pictures in depression. J Abnorm Psychol (2004) 113:654–60. 10.1037/0021-843X.113.4.654 15535797

[4] Hechtman LA Raila H Chiao J Gruber J. Positive emotion regulation and psychopathology: a transdiagnostic cultural neuroscience approach. J Exp Psychopathol (2013) 4:1–27. 10.5127/jep.030412 24812583 PMC4011185

[5] Holmes EA Lang TJ Shah DM. Developing interpretation bias modification as a “cognitive vaccine” for depressed mood: imagining positive events makes you feel better than thinking about them verbally. J Abnorm Psychol (2009) 118:76–88. 10.1037/a0012590 19222316

[6] Kang Y Gruber J. Harnessing happiness? Uncontrollable positive emotion in bipolar disorder, major depression, and healthy adults. Emotion (2013) 13:290–301. 10.1037/a0030780 23205524

[7] American Psychiatric Association. Diagnostic and Statistical Manual of Mental Disorders. 5th ed Arlington, VA: American Psychiatric Association (2013).

[8] Geschwind N Peeters F Drukker M van Os J Wichers M. Mindfulness training increases momentary positive emotions and reward experience in adults vulnerable to depression: a randomized controlled trial. J Consult Clin Psychol (2011) 79:618–28. 10.1037/a0024595 21767001

[9] Holmes EA Lang TJ Deeprose C. Mental imagery and emotion in treatment across disorders: using the example of depression. Cogn Behav Ther (2009) 38(Suppl 1):21–8. 10.1080/16506070902980729 19697177

[10] Werner-Seidler A Moulds ML. Autobiographical memory characteristics in depression vulnerability: formerly depressed individuals recall less vivid positive memories. Cogn Emot (2011) 25:1087–103. 10.1080/02699931.2010.531007 21895571

[11] Joormann J Siemer M Gotlib IH. Mood regulation in depression: differential effects of distraction and recall of happy memories on sad mood. J Abnorm Psychol (2007) 116:484–90. 10.1037/0021-843X.116.3.484 17696704

[12] Holmes EA Mathews A Mental imagery and emotion: a special relationship? Emotion (2005) 5:489–97 10.1037/1528-3542.5.4.489 16366752

[13] Moberly NJ Watkins ER. Processing mode influences the relationship between trait rumination and emotional vulnerability. Behav Ther (2006) 37:281–91. 10.1016/j.beth.2006.02.003 16942979

[14] Watkins E Moberly NJ Moulds ML. Processing mode causally influences emotional reactivity: distinct effects of abstract versus concrete construal on emotional response. Emotion (2008) 8:364–78. 10.1037/1528-3542.8.3.364 18540752 PMC2672048

[15] Holmes EA Mathews A Dalgleish T Mackintosh B. Positive interpretation training: effects of mental imagery versus verbal training on positive mood. Behav Ther (2006) 37:237–47. 10.1016/j.beth.2006.02.002 16942975

[16] Werner-Seidler A Moulds ML. Mood repair and processing mode in depression. Emotion (2012) 12:470–8. 10.1037/a0025984 22023367

[17] Rohrbacher H Blackwell SE Holmes EA Reinecke A. Optimizing the ingredients for imagery-based interpretation bias modification for depressed mood: is self-generation more effective than imagination alone? J Affect Disord (2014) 152-154:212–8. 10.1016/j.jad.2013.09.013 24113076 PMC3878375

[18] Holmes EA Geddes JR Colom F Goodwin GM. Mental imagery as an emotional amplifier: application to bipolar disorder. Behav Res Ther (2008) 46:1251–8. 10.1016/j.brat.2008.09.005 18990364

[19] Bryant FB Smart CM King SP Using the past to enhance the present: boosting happiness through positive reminiscence. J Happiness Stud (2005) 6:227–60 10.1007/s10902-005-3889-4

[20] Werner-Seidler A Moulds ML. Recalling positive self-defining memories in depression: the impact of processing mode. Memory (2014) 22:525–35. 10.1080/09658211.2013.801494 23758472

[21] Feldman GC Joormann J Johnson SL. Responses to positive affect: a self-report measure of rumination and dampening. Cognit Ther Res (2008) 32:507–25. 10.1007/s10608-006-9083-0 20360998 PMC2847784

[22] Raes F Daems K Feldman GC Johnson SL Van Gucht D A psychometric evaluation of the Dutch version of the responses to positive affect questionnaire. Psychol Belg (2009) 49:293–310 10.5334/pb-49-4-293

[23] Raes F Smets J Nelis S Schoofs H. Dampening of positive affect prospectively predicts depressive symptoms in non-clinical samples. Cogn Emot (2012) 26:75–82. 10.1080/02699931.2011.555474 21756217

[24] Marigold DC Holmes JG Ross M. More than words: reframing compliments from romantic partners fosters security in low self-esteem individuals. J Pers Soc Psychol (2007) 92:232–48. 10.1037/0022-3514.92.2.232 17279847

[25] Watkins E. Adaptive and maladaptive ruminative self-focus during emotional processing. Behav Res Ther (2004) 42:1037–52. 10.1016/j.brat.2004.01.009 15325900

[26] Beck AT Steer RA Brown GK Beck Depression Inventory. 2nd ed San Antonio, TX: The Psychological Corporation (1996).

[27] Van der Does AJW Handleiding bij de Nederlandse bewerking van de BDI-II [Manual of the Dutch version of the BDI-II]. San Antonio, TX: The Psychological Corporation (2002). Lisse: Swets Test Publishers.

[28] Treynor W Gonzalez R Nolen-Hoeksema S Rumination reconsidered: a psychometric analysis. Cognit Ther Res (2003) 27:247–59 10.1023/A:1023910315561

[29] Williams JMG Broadbent K Autobiographical memory in suicide attempters. J Abnorm Psychol (1986) 95:144–9 10.1037/0021-843X.95.2.144 3711438

[30] Debeer E Hermans D Raes F. Associations between components of rumination and autobiographical memory specificity as measured by a minimal instructions autobiographical memory test. Memory (2009) 17:892–903. 10.1080/09658210903376243 19882439

[31] Bylsma LM Taylor-Clift A Rottenberg J. Emotional reactivity to daily events in major and minor depression. J Abnorm Psychol (2011) 120:155–67. 10.1037/a0021662 21319928

[32] Peeters F Nicolson NA Berkhof J Delespaul P deVries M. Effects of daily events on mood states in major depressive disorder. J Abnorm Psychol (2003) 112:203–11. 10.1037/0021-843X.112.2.203 12784829

[33] Beblo T Fernando S Klocke S Griepenstroh J Aschenbrenner S Driessen M. Increased suppression of negative and positive emotions in major depression. J Affect Disord (2012) 141:474–9. 10.1016/j.jad.2012.03.019 22483953

[34] D’Argembeau A Van der Linden M. Phenomenal characteristics associated with projecting oneself back into the past and forward into the future: influence of valence and temporal distance. Conscious Cogn (2004) 13:844–58. 10.1016/j.concog.2004.07.007 15522635

[35] Strauman TJ Higgins ET. Automatic activation of self-discrepancies and emotional syndromes: when cognitive structures influence affect. J Pers Soc Psychol (1987) 53:1004–14. 10.1037/0022-3514.53.6.1004 3694448

[36] Strack F Schwarz N Gschneidinger E Happiness and reminiscing: the role of time perspective, affect, and mode of thinking. J Pers Soc Psychol (1985) 49:1460–9 10.1037/0022-3514.49.6.1460

[37] Joormann J Siemer M. Memory accessibility, mood regulation, and dysphoria: difficulties in repairing sad mood with happy memories? J Abnorm Psychol (2004) 113:179–88. 10.1037/0021-843X.113.2.179 15122938

[38] Conway MA. Sensory-perceptual episodic memory and its context: autobiographical memory. Philos Trans R Soc Lond B Biol Sci (2001) 356:1375–84. 10.1098/rstb.2001.0940 11571029 PMC1088521

[39] Tulving E Episodic memory: from mind to brain. Annu Rev Psychol (2002) 53:1–25 10.1146/annurev.psych.53.100901.135114 11752477

[40] Nigro G Neisser U Point of view in personal memories. Cogn Psychol (1983) 15:467–82 10.1016/0010-0285(83)90016-6

[41] Libby LK Shaeffer EM Eibach RP. Seeing meaning in action: a bidirectional link between visual perspective and action identification level. J Exp Psychol (2009) 138:503–16. 10.1037/a0016795 19883133

[42] Libby LK Eibach RP Self-enhancement or self-coherence? Why people shift visual perspective in mental images of the personal past and future. Pers Soc Psychol Bull (2011) 37:714–26 10.1177/0146167211400207 21487115

[43] Holmes EA Coughtrey AE Connor A. Looking at or through rose-tinted glasses? Imagery perspective and positive mood. Emotion (2008) 8:875–9. 10.1037/a0013617 19102599

[44] Kuyken W Howell R Facets of autobiographical memory in adolescents with major depressive disorder and never-depressed controls. Cogn Emot (2006) 20:466–87 10.1080/02699930500342639 26529216

[45] Kuyken W Moulds ML. Remembering as an observer: how is autobiographical memory retrieval vantage perspective linked to depression? Memory (2009) 17:624–34. 10.1080/09658210902984526 19536690

[46] Nelis S Debeer E Holmes E Raes F. Dysphoric students show higher use of the observer perspective in their retrieval of positive versus negative autobiographical memories. Memory (2013) 21:423–30. 10.1080/09658211.2012.730530 23083015 PMC3746460

[47] Lovibond SH Lovibond PF Manual for the Depression Anxiety Stress Scales. 2nd ed Sydney, NSW: Psychology Foundation (1995).

[48] de Beurs E Van Dyck R Marquenie LA Lange A Blonk RWB De DASS: een vragenlijst voor het meten van depressie, angst en stress. [The DASS: a questionnaire for the measurement of depression, anxiety, and stress]. Gedragstherapie (2001) 34:35–53.

[49] Rice HJ Rubin DC. I can see it both ways: first- and third-person visual perspectives at retrieval. Conscious Cogn (2009) 18:877–90. 10.1016/j.concog.2009.07.004.I 19692271 PMC2784183

[50] Raes F Watkins ER Williams JMG Hermans D. Non-ruminative processing reduces overgeneral autobiographical memory retrieval in students. Behav Res Ther (2008) 46:748–56. 10.1016/j.brat.2008.03.003 18456242

[51] Hetherington K Moulds ML. Does mode of processing during a positive experience have consequences for affect? J Behav Ther Exp Psychiatry (2013) 44:165–71. 10.1016/j.jbtep.2012.10.002 23207963

[52] Marigold DC Holmes JG Ross M Fostering relationship resilience: an intervention for low self-esteem individuals. J Exp Soc Psychol (2010) 46:624–30 10.1016/j.jesp.2010.02.011

[53] Raes F Williams JMG Hermans D. Reducing cognitive vulnerability to depression: a preliminary investigation of MEmory Specificity Training (MEST) in inpatients with depressive symptomatology. J Behav Ther Exp Psychiatry (2009) 40:24–38. 10.1016/j.jbtep.2008.03.001 18407245

[54] Blackwell SE Browning M Mathews A Pictet A Welch J Davies J Positive imagery-based cognitive bias modification as a web-based treatment tool for depressed adults: a randomized controlled trial. Clin Psychol Sci (2015) 3:91–111 10.1177/2167702614560746 PMC4359210 25984421

[55] Lang TJ Blackwell SE Harmer CJ Davison P Holmes EA. Cognitive bias modification using mental imagery for depression: developing a novel computerized intervention to change negative thinking styles. Eur J Pers (2012) 157:145–57. 10.1002/per 23316101 PMC3532611

[56] Williams AD Blackwell SE Mackenzie A Holmes EA Andrews G. Combining imagination and reason in the treatment of depression: a randomized controlled trial of internet-based cognitive-bias modification and internet-CBT for depression. J Consult Clin Psychol (2013) 81:793–9. 10.1037/a0033247 23750459 PMC3780629

[57] Grol M Koster EHW Bruyneel L De Raedt R. Effects of positive mood on attention broadening for self-related information. Psychol Res (2014) 78:566–73. 10.1007/s00426-013-0508-6 23975116

[58] Pictet A Coughtrey AE Mathews A Holmes EA. Fishing for happiness: the effects of generating positive imagery on mood and behaviour. Behav Res Ther (2011) 49:885–91. 10.1016/j.brat.2011.10.003 22032936 PMC3240747

[59] LeMoult J Arditte KA D’Avanzato C Joormann J. State rumination: associations with emotional stress reactivity and attention biases. J Exp Psychopathol (2013) 4:471–84. 10.5127/jep.029112 25431652 PMC4243309

